# Suppression of microRNA-9-5p rescues learning and memory in chronic cerebral hypoperfusion rats model

**DOI:** 10.18632/oncotarget.22415

**Published:** 2017-11-11

**Authors:** Na Wei, Kai Zheng, Rui Xue, Sheng-Li Ma, Hua-Yan Ren, Hui-Fen Huang, Wei-Wei Wang, Jing-Jing Xu, Kui-Sheng Chen

**Affiliations:** ^1^ Department of Pathology, The First Affiliated Hospital of Zhengzhou University, Zhengzhou 450002, People's Republic of China; ^2^ Henan Key Laboratory of Tumor Pathology, Zhengzhou 450002, People's Republic of China; ^3^ Department of Pathology, School of Basic Medicine, Zhengzhou University, Zhengzhou 450002, People's Republic of China; ^4^ Department of Geriatrics, Tongji Hospital, Tongji Medical College, Huazhong University of Science and Technology, Wuhan 430030, China; ^5^ Medical Research Center, The First Affiliated Hospital of Zhengzhou University, Zhengzhou 450002, People's Republic of China; ^6^ Department of Emergency, The First Affiliated Hospital of Zhengzhou University, Zhengzhou 450002, People's Republic of China

**Keywords:** miR-9-5p, memory, chronic cerebral hypoperfusion, long-term potentiation

## Abstract

Chronic cerebral hypoperfusion has been associated with cognitive impairment in dementias, such as Alzheimer's disease (AD) and vascular disease (VaD), the two most common neurodegenerative diseases in aged people. However, the effective therapeutic approaches for both AD and VaD are still missing. MicroRNAs (miRNAs) are small non-coding RNAs that play important roles in the epigenetic regulation in many neurological disorders; the critical roles of miRNAderegulation had been implicated in both AD and VaD. In the current study, we reported that miR-9-5p is elevated in the serum and cerebrospinalfluid of patientswith VaD. The miR-9-5p wasalso increased in both the hippocampus and cortex of rats with 2-vessel occlusionsurgery. Furthermore, application ofmiR-9-5p antagomirs attenuated the memory impairments in rats with 2-vessel occlusion surgery both in the Morris water maze and inhibitory avoidance step-down tasks. Furthermore, miR-9-5p antagomirs reducedthe inhibition oflong-term potentiation and loss of dendritic spines in chronic cerebral hypoperfusionrats. Additionally, the cholinergic neuronal function was rescued by miR-9-5p antagomirs, as well as the neuronal loss and the oxidative stress. We concluded that miR-9-5p inhibition may be a potential therapeutic target for the memory impairments caused by chronic cerebral hypoperfusion.

## INTRODUCTION

Dementia is a broad category of brain diseases that cause a long term and gradualdecrement in emotionsand memory, which is great enough to reduce a person's ability to perform everyday activities [1]. There are severaltypes of dementia, including Alzheimer's disease (AD), vascular dementia (VaD), Lewy dody dementia (LBD), andfrontotemporal dementia (FTD) among others. Among them, AD and VaD are the two most prevalent types, which account for more than 80% of the dementia cases [2].

In the brain of AD and VaD, the blood flow was reported to be reduced at an early stageand to be directly correlated with cognitive measures [3]. It is known that the flow of blood delivers the essential oxygen and nutrients to the brain cells and plays animportant rolein the maintenanceof normal thinking, learning, and memory [4]. The dramaticbloodflow reduction in patientswith AD orVaD may potentially lead to brain cell damage and cognitive decline [5, 6]. Indeed, as a result of vascular risk factors such as hypertension, diabetes mellitus, hypercholesterolemia, and smoking, chronic cerebral hypoperfusion (CCH) is a common vascular component among AD risk factors [7]. In VaD, the deepbrain areas, particularly white matter areas, suffer from chronic and moderate ischemia, which is a state representing one of the physiopathological mechanisms of damage [8]. Consistent with this, the microvesselsin the AD and VaDbrainsare frequently narrowed, degenerated, and amyloid-laden, suggesting a pivotal role of cerebrovascularfactors in both AD and VaD [9, 10]. Additionally, numerous studies have suggested that CCH might promote neurodegenerationthrough neuronal energy failure, production of reactive oxygen species, and proinflammatory cytokines by activated microglial cells that, in turn, damage the neuronal cells and contribute to white matter lesions [11].

MicroRNAs (miRNAs) are small (-22 nucleotides), endogenous noncoding RNA molecules that play important roles in diverse biological processes [12]. The mature miRNAis incorporated into the miRNA-induced silencing complex (miRISC) and then guides it to target sequences by recognizing their target sites located in 3’UTRs *via* incomplete base-pairing. The binding of miRNAwith its targets always leads tomRNAdestabilization or translational repression of the target genes [13, 14]. The aberrant regulation of miRNAshad been reported in both AD and VaD brains [15, 16]. Among them, miR-9, which is a highly conserved miRNAlocated on chromosome 3 in the mouse genome, is of particularinterest. Previous studies suggested that miR-9 is enriched in the brain, especially during development [17]. In AD brains, the level of miR-9 is increased in the temporal lobes, neocortex, and hippocampalregions when compared with age-matched healthy adults [18, 19]. However, the role of miR-9 in the progression of memory impairment induced by vascular factors hasnot been studied yet.

In this study, we reported that miR-9-5p is upregulatedin both the serum and cerebrospinalfluid of patients with VaD and in the hippocampus of CCH rats. Furthermore, reduction of miR-9-5p by antagomirsrescued the learning and memory ability, synaptic plasticity, dendritic spines, cholinergic neurons, oxidative stress level, and neuronal loss induced by CCH.

## RESULTS

### miR-9-5p is upregulated in patients with VaD and CCHrats

**Figure 1:**
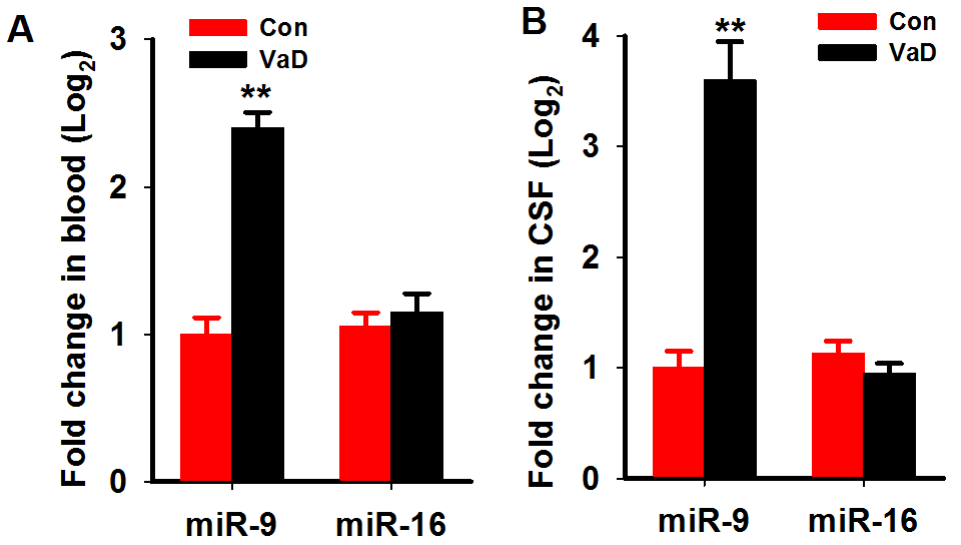
MiR-9-5p is upregulated in the serum and CSF of patientswith VaD The serum and CSFof patients with VaD and age-matched controls were collected as described in Methods. The level of miR-9-5p and miR-16 in the serum **(A)** and CSF **(B)** were detected by Q-PCR. CSF, cerebrospinal fluid; VaD, vascular dementia; ^**^, *P*<0.01, compared with the controls.

**Figure 2:**
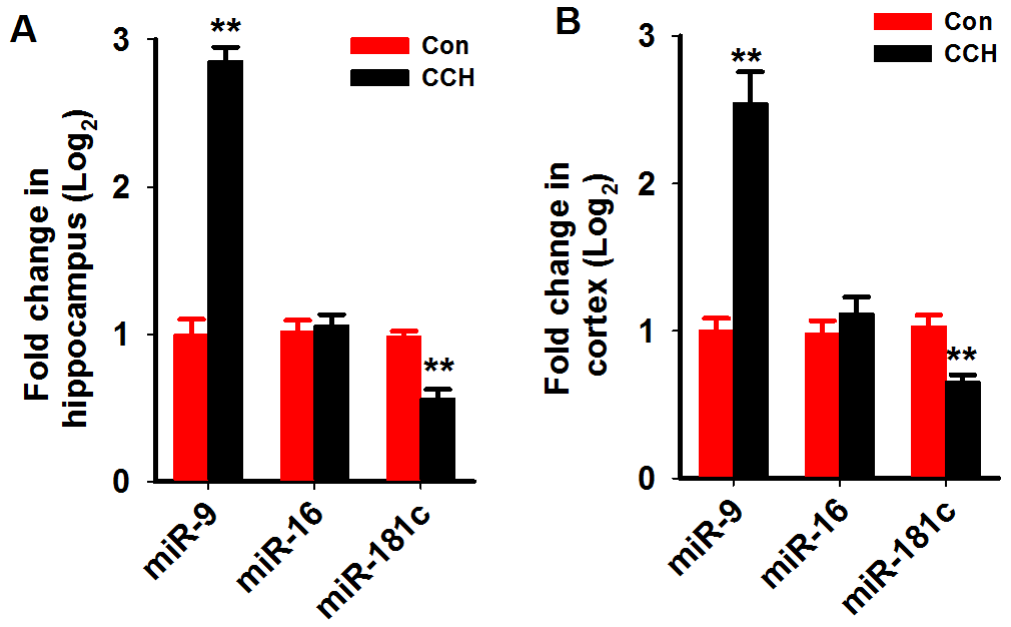
MiR-9-5p is increased in the hippocampus and cortex of CCH rats Three months after the 2VO surgery, the hippocampus **(A)** and cortex **(B)** were extracted and the RNA wasisolated. The levels of miR-9-5p and miR-16 were evaluated by Q-PCR by using specific primers. 2VO, 2-vessel occlusion;CCH, chronic cerebral hypoperfusion; ^**^, *P*<0.01, compared with Conrats.

We first analyzed the miR-9-5p level in the serum and cerebrospinalfluid (CSF) of patients with VaD as described above. We found that in the serum of patientswith VaD, the level of miR-9-5p increased to about 2.4 folds of age-matched controls (Figure 1A). A more prominent increment was found in the CSF samples (Figure 1B). As a miRNAcontrol, the level of miR-16 wasnot changed in boththe serum and CSF (Figure 1A, 1B). We further examined the miR-9-5p level in the hippocampi and corticestissues of rats at 3 monthafter the 2VO surgery. As expected, we detected an increase inmiR-9-5plevels both in the hippocampus andcortex ofthe CCH rats (Figure 2A, 2B). Similarly to our findings in patients, the levels of miR-16 werenot changed but the levels of miR-181c were reducedin the CCH rats (Figure 2A, 2B). These data strongly suggested that miR-9-5p is upregulatedin patients with VaD and CCH rats.

### Reduction of miR-9-5p *in vivo* rescues learning and memory impairments in CCH rats

**Figure 3:**
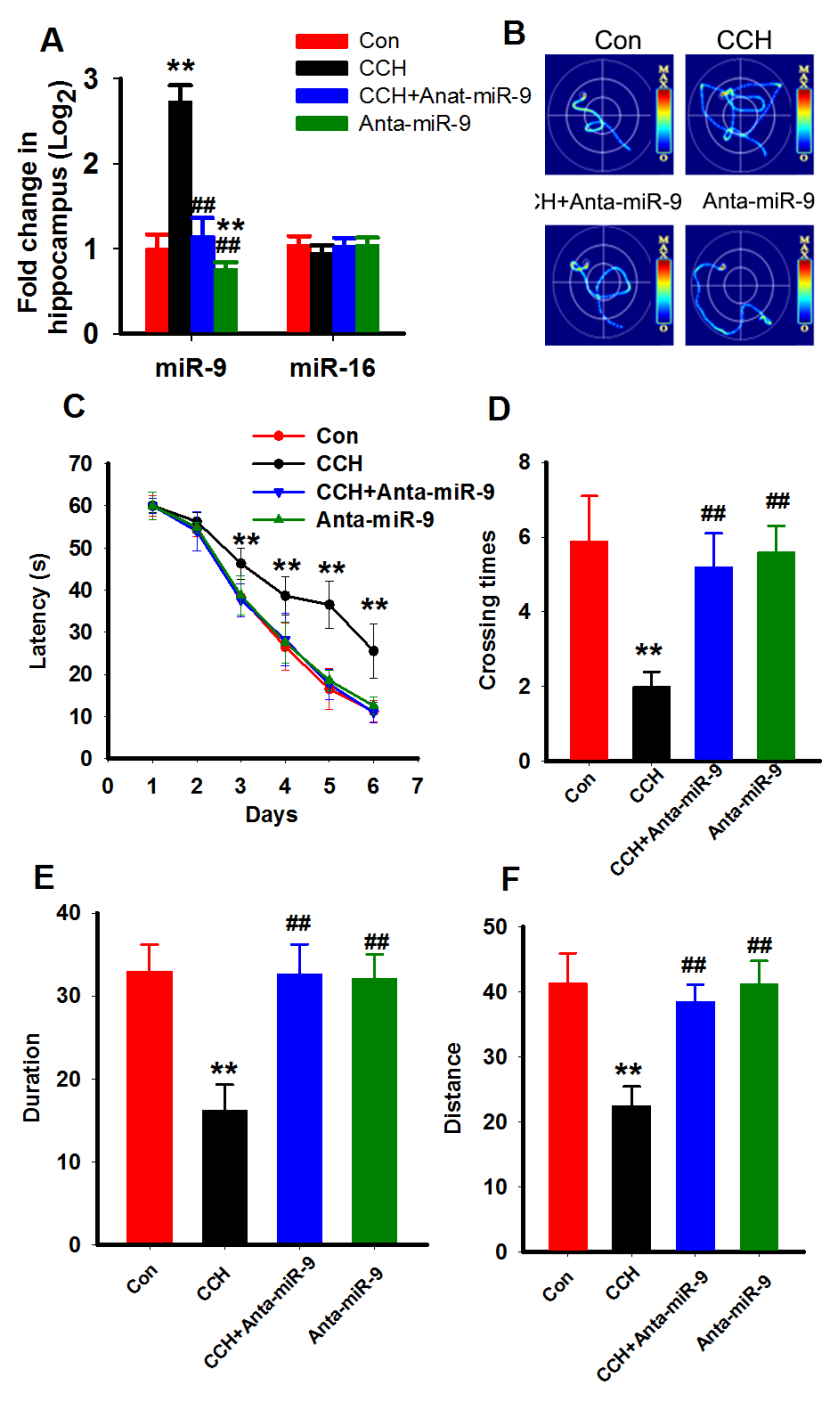
Inhibition of miR-9-5p rescued the spatial learning memory impairments of CCH rats Three months after the surgery, Morris water maze was applied to evaluate the spatial memory of rats. **(A)** The miR-9-5p and miR-16 levels in different treated rats. ^**^ *P*<0.01, compared to control rats. ^##^*P*<0.01, compared withCCH rats. **(B)** The representative escape traces for different groups in the final training day. **(C)** The latency during the six-days training task in the Morris water maze. (D–F) The total crossing times **(D)**, the total time spent in the target quadrant **(E)**, and the total swimming distances during the probe trial **(F)**. CCH, chronic cerebral hypoperfusion;^**^P<0.01, compared with sham rats; ^##^*P*<0.01, compared with CCH rats.

To understand the effects of miR-9-5p increment in the pathogenesis of VaD, we artificially suppressedthe expression of miR-9-5p in the brains of theCCH ratswith rno-miR-9-5p antagomirat 3 months after the surgery (Figure 3A). Subsequently, 2 weeks later, the spatial learning and memory abilities of the rats were evaluated with the MWM task. The swimmingtracks of the rats revealedthat the sham rats reached the platform inless than 20 s by using a direct searching strategy, while the CCH rats tookover 40 s using a random searching strategy. Treatingthe rats with anta-miR-9-5p improved the searching strategy (Figure 3B). On the first 2trainingdays, we did notdetectany significant difference among the groups. Beginning on training day 3, the escape latency of the CCH group was longer than that of the sham group, and treatment with anta-miR-9-5p significantly decreased the latencyin the CCH+Anta-miR-9-5pgroup comparedwith the CCH group (Figure 3C). In the probe trial, rats in the CCH group displayed less crossing times to the platform region and less duration and distance in the target quadrantthan the sham group, while treatment with Anta-miR-9-5p improved thesemeasuresinCCH rats (Figure 3D-3F). No obvious difference was detectedbetween the Conand Anta-miR-9-5prats (Figure 3D-3F).

**Figure 4:**
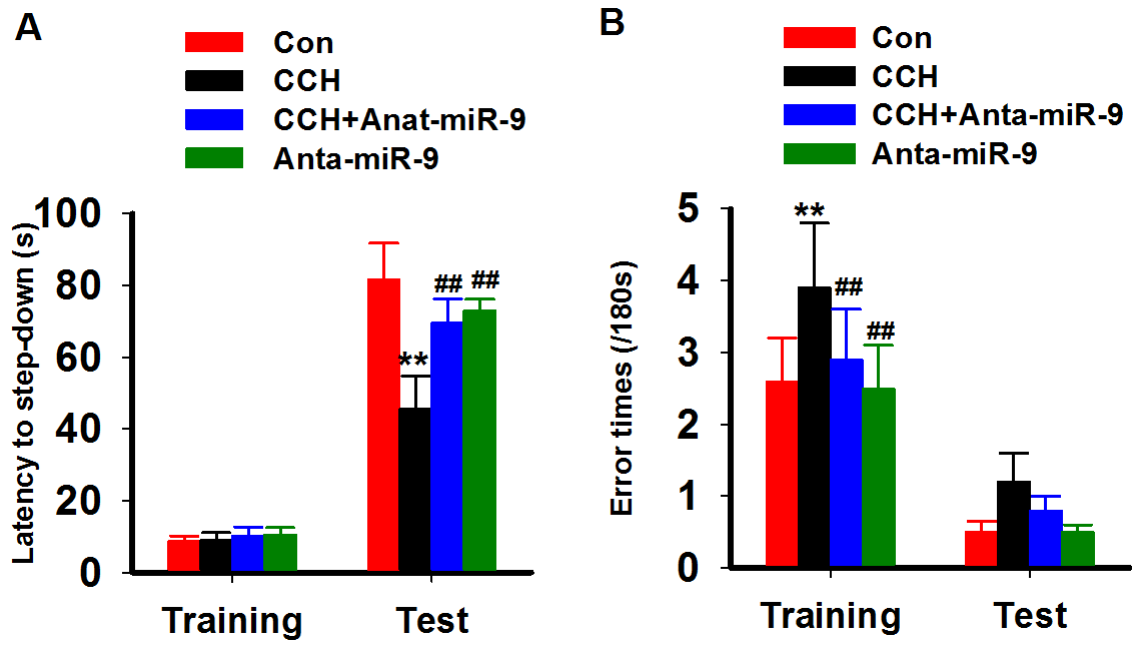
Inhibition of miR-9-5p rescued the fear memory impairments of CCH rats The inhibitory avoidance tasks are applied to analyze fear memory of rats. After training, long-term memory test are carried at 24 h later. The step-down latency **(A)** and error times **(B)** were recorded. CCH, chronic cerebral hypoperfusion; ^**^P<0.01, compared with Conrats; ^##^*P*<0.01, compared with CCH rats.

We used the step-down inhibitory avoidance test to evaluate the emotional cognition of the rats. During training, nosignificant differences were found in the step-down latency among the groups. In the test period, CCH rats showed obviously a shorter latency than control rats, whileanta-miR-9-5pextended the latency of CCH rats (Figure 4A). We also calculated the errors in both the training and test periods and found that CCH rats made more errors in both stages, while anta-miR-9-5p reduced the number of errors (Figure 4B). No significant differences were found between the Conand Anta-miR-9-5p groups. The above results indicated that suppression of miR-9-5p is able to reverse the memory deficits in CCH rats.

### Reduction of miR-9-5p *in vivo* rescues the synaptic impairments in CCH rats

**Figure 5:**
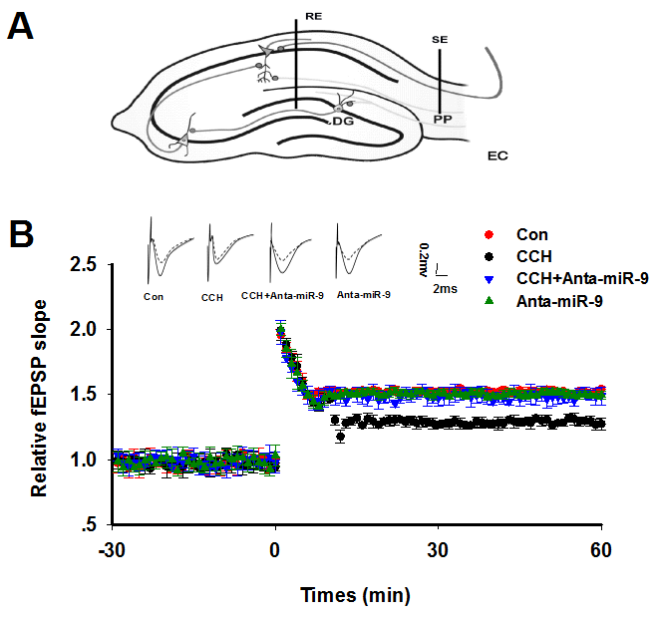
Inhibition of miR-9-5p rescued the LTP inhibition in CCH rats **(A)** The diagram for LTP recording. RE: recording electrode; SE: stimulating electrode; PP: perforant path; DG: dentate gyrus. **(B)** Upper panel: The representative analog traces of evoked potentials before (dashline) and after (solid line) HFS with different treatmentsare shown. Lower panel: The alterations of LTP represented by normalized slope of EPSP were recorded. The electrophysiology recording was started 30 min (-30), and the HFS was added at 0 min. The data represent means± standard error of the mean. CCH, chronic cerebral hypoperfusion; EPSP, excitatory postsynaptic potential; HFS, high frequency stimulation; LTP, long-term potentiation.

**Figure 6:**
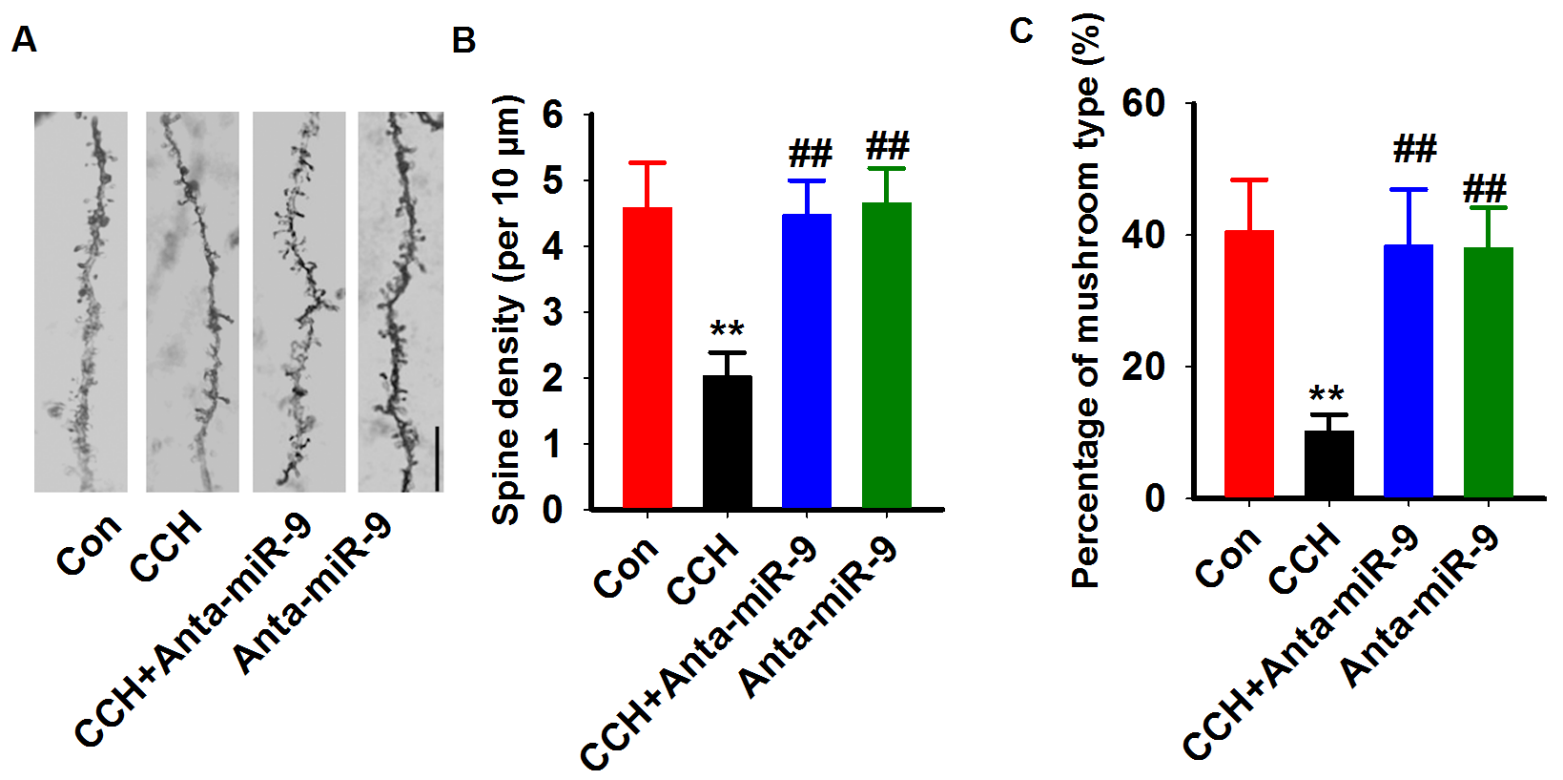
Inhibition of miR-9-5p rescued the dendritic spines abnormalities in CCH rats **(A)** Representative images of dendritic spine were captured from Golgi staining of DG region. Bar=20μm. **(B, C)** The quantitative analysis of the density of spines (B) and the percentage of mushroom types (C) were carried. CCH, chronic cerebral hypoperfusion; ^**^P< 0.01*vs.*Congroup; ^##^*P*< 0.01*vs.* CCH group.

Previous studies suggested that synaptic plasticity is the basis for learning and memory. We examined synaptic plasticity by evaluating LTP modificationsin the hippocampus of CCH rats. We found that the CCH rats displayed decreased slope of EPSP, which were lower than the ratsof the Sham group. Treatment withanta-miR-9-5pelevated the declined LTP induced by CCH (Figure 5A, 5B). No significant differences were found between the Conand Anta-miR-9-5p groups. Since dendritic spines are animportant morphological basis for synaptic plasticity, we also examined the dendritic spines in the dentate gyrususingGolgistaining. As expected, we found that CCH significantly decreased not only the density of dendritic spines but also the percentage of mushroom-type spines (Figure 6A-6C). The pre-administrated ofanta-miR-9-5p strongly improved theCCH-inducedspinogenesisinhibition. These results strongly implied that the *in vivo* suppression of miR-9-5p is able to reverse the synaptic impairments in CCH rats.

### Reduction of miR-9-5p *in vivo* reduces the cholinergic system in CCH rats

**Figure 7:**
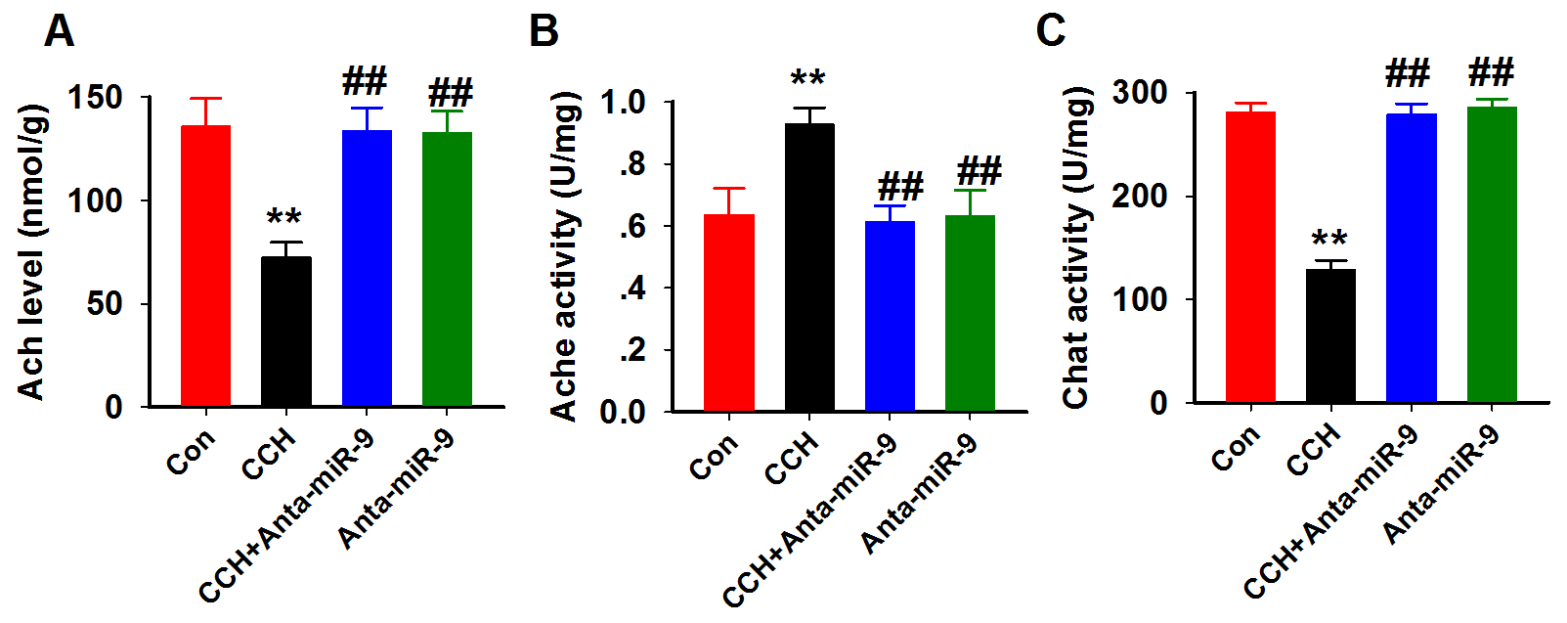
Inhibition of miR-9-5p rescued the cholinergic dysfunction in CCH rats The cholinergic system function was assayed using ACh **(A)**, AChE **(B)** and ChAT **(C)** kits as described above. CCH, chronic cerebral hypoperfusion; ^**^P< 0.01*vs.*Congroup; ^##^*P*< 0.01*vs.* CCH group.

Previous studies suggested that the central cholinergic system dysfunction is involved in memory impairments inCCH rats [20]. Therefore, we measured the effect of suppression of miR-9-5p on AChlevelsand activity of AChE and ChAT in the hippocampus of CCH rats. Consistent withprevious reports [21], CCH resulted in a significant decrease of ACh levels, increased AChE activity, and a dramatic reduction of ChAT activity in the hippocampus, which confirmed the impaired cholinergic function in CCH. Moreover, we found that suppression of miR-9-5pby anta-miR-9-5p in CCH rats elevated AChlevelsand ChAT activity, butdecreased AChE activity. No significant differences were found between the Conand anta-miR-9-5p groups (Figure 7A-7C). These data support the behavioral results and suggest that reduction of miR-9-5p may exert its neuroprotective effectsby restoring thecholinergic function.

### Reduction of miR-9-5p *in vivo* reduces the neuronal loss in CCH rats

**Figure 8:**
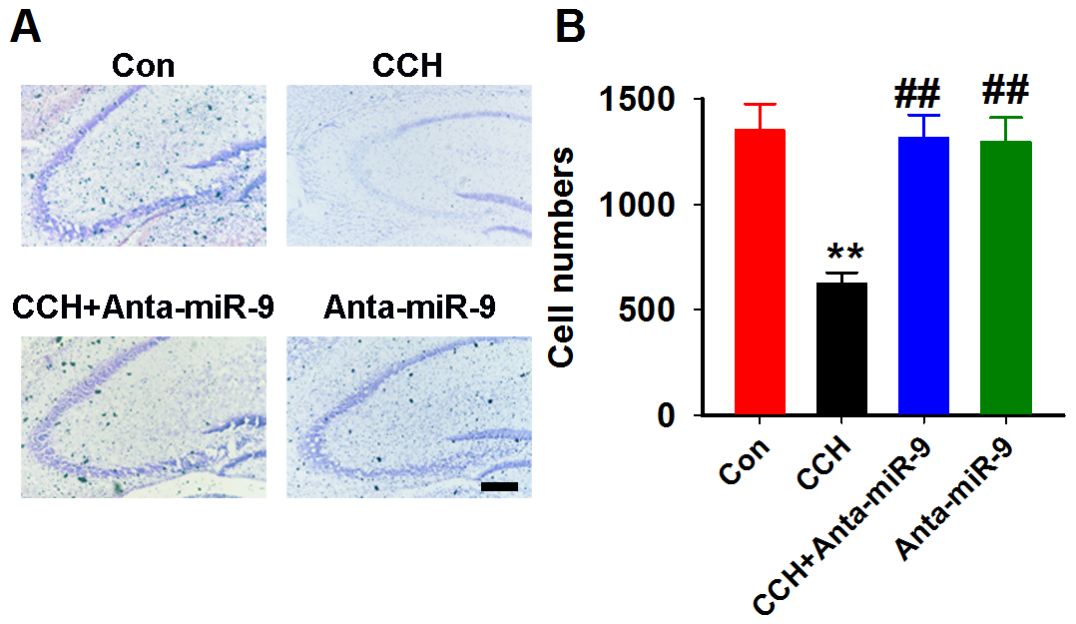
Inhibition of miR-9-5p restored the neuronal loss in CCH rats **(A)** Arepresentative image ofthe hippocampus; scale bar=100μm. **(B)** The quantitative analysis for the whole hippocampus. CCH, chronic cerebral hypoperfusion; ^**^P< 0.01*vs.*Congroup; ^##^*P*< 0.01*vs.* CCH group. N=16 slices from 4 rats.

It was reported that neuronal loss resulting from apoptotic or necroticneuronal cell death is a common feature of VaD [22]. We also examined whether the reduction of miR-9-5p *in vivo* could reverse the neuronal loss in CCH rats by using the Nissl staining. Quantitative analysis suggested that CCH induces a dramatic neuronal loss in the hippocampuswhile pre-administrationofanta-miR-9-5p strongly restored the number ofneurons in CCH rats (Figure 8A, 8B).

### Reduction of miR-9-5p inhibits the oxidative stress level in CCH rats

**Figure 9:**
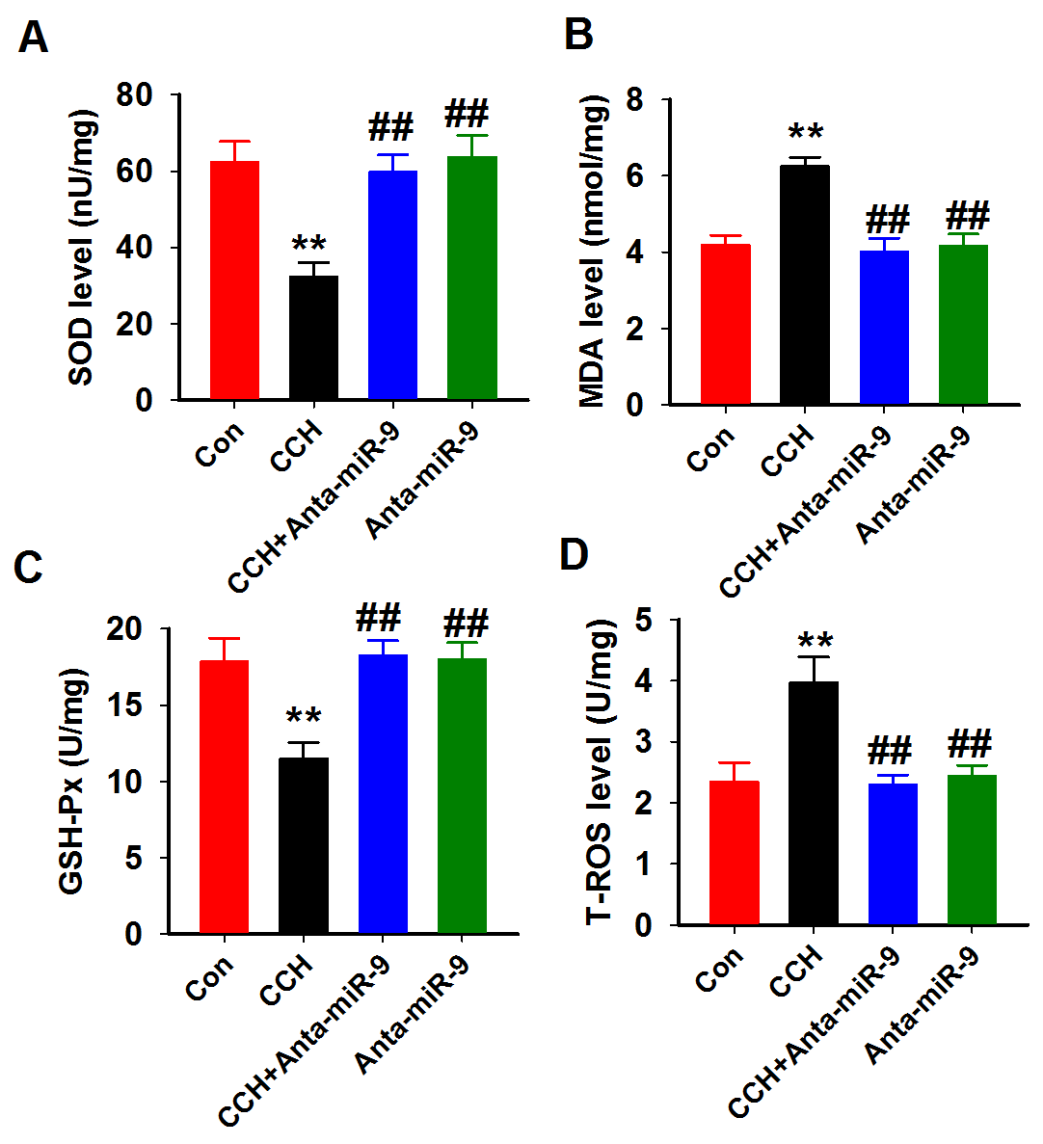
Inhibition of miR-9-5p attenuated the oxidative stress in CCH rats Four oxidative stress parameters were assayed in the hippocampus homogenates from different groups by using SOD **(A)**, MDA **(B)**, GSH-Px **(C)** and T-ROS **(D)** kits described above. CCH, chronic cerebral hypoperfusion; GSH-Px, glutathione peroxidase; MDA, malonic dialdehyde; SOD, superoxide dismutase; T-ROS, reactive oxygen species ^**^P< 0.01 *vs.*control group; ^##^*P*< 0.01 *vs.* CCH group.

As the oxidative stress was reported to induce neuronal loss in CCH rats [23], we examined the activities of SOD, GSH-px, and the levelsof MDA and T-ROS in the hippocampal homogenates of CCH rats. As predicted, the SOD and GSH-px activities werereduced, but the levels of MDA and T-ROS were increased compared with controls. Administration of anta-miR-9-5p significantly restored the activities of SOD and GSH-pxand decreased the levels of MDA and T-ROS in the CCH rats (Figure 9A-9D). These data suggested that suppression of miR-9-5p inhibits the oxidative stress level in CCH rats.

## DISCUSSION

VaD is thought to be a neurodegenerative disorder caused by multiple vasculotoxic and neurotoxic effects due to diminished cerebral blood flow, which leads to hypoxia and altered permeability of the blood-brain barrier. Severalpathological events have been suggested to be the prime risk factorsfor VaD, such as stroke, cerebral hemorrhage, trauma, chronic diseases likeatherosclerosis, large and small vessel disease, and cardioembolic disease [24]. To date, the genetic basis of VaDisnot well defined. MiRNAs belong to a class of endogenous, stable, non-coding RNA molecules involved in the regulation of target gene expressionat the post-transcriptional levelby either the degradation of the RNA or translational arrest [25]. Several miRNAs are expressed specifically in the CNS, where some are proposed to function in neuronal activities such as neurite outgrowth and synapse formation [26], and in neurodegenerative diseases [27, 28], including VaD [15].

Our results demonstrated that miR-9-5p is critically involved in VaD and that blocking miR-9-5p is sufficientto rescue the learning and memory impairments observed ina VaDrat model. The miR-9-5p gene is evolutionary well conserved. To date, several functional studies on miR-9 have emphasizeditsrole in neuronal development and neurogenesis [29]. For example, miR-9 knock-out mice, in which both miR-9-5p and miR-9-3p are reduced, displayed obvious defects in neurogenesisand abnormal telencephalicstructures [30]. It should be mentioned that miR-9-5p and miR-9-3p are abundantly expressed not only in neural progenitors but also in postmitotic neurons [17, 31];hence the importance of studying the potential role of miR-9 in adulthood and neurological diseases. Indeed, in the postmortem brains of patients withHuntington's disease, the level of miR-9 was decreased [32]. In brains from patients withAD, the level of miR-9wasincreased in the temporal lobes, neocortex, and hippocampalregions when compared withage-matched healthy adults [18, 19]. In the current study, we demonstratedan abnormal upregulation of miR-9-5p in both the serum and CSF of patientswith VaD, suggesting thepotential role of miR-9-5p in VaD progression.

Synaptic plasticity and dendritic spines are the basis for learning and memory. Dendritic spines serve as a storage site for synaptic strength and help transmit electrical signals to the neuron's cell body [33]. Spines constitute a specialized compartment that contains multiple signaling complexes, which play important roles in synaptic transmission [34]. The disruption of synapses playsan important role in VaD [35]. Thus, the preservation of dendritic spines predictably restores synaptic plasticity, as well as learning and memory. Recently, in primary culture neurons, Giusti et al. [48] showed that the inhibition of miR-9-5p using the sponge technique impairs dendritic growth and excitatory synaptic transmission. Interestingly, the miR-9-3p sponge had no effect on dendritic growth of cultured neurons [36]. In an independent study, asynergistic effectof miR-9 and miR-124 has been reportedin the regulation of dendritic branching via the AKT/GSK3β pathway by targeting the Rap GTP-binding proteinRap2a [37]. In our study, we found that the administration of miR-9-5p antagomir restores the dendritic spines loss and the synaptic plasticity inhibition in the CCH rats. Moreover, miR-9-5p antagomir did not alter the dendritic spines and LTP in normal rats. These data suggested a discrepancy inthe *in vivo* and *in vitro* roles of miR-9-5p in dendritic spines and synaptic plasticity. We propose that such differences may be the result of the more complicated environmentin the brain compared with that of cultured neurons.

## MATERIALS AND METHODS

### Patients and selection criteria

A total of 25 patients with VaD (14 men and 11 women) whose diagnoses were confirmed using the National Institute of Neurological Disorders and Stroke-Association Internationale pour la Recherche et l’Enseignement en Neurosciences (NINDS-AIREN) criteria for VaD [38]. The Mini–Mental State Examinationscores of all patients were less than 25. A total of 22 healthy age-matched participants (12 men and 10 women) were recruited from the outpatient setting and served as the control group. The control group was selected from the Medical Examination Centre of the First Affiliated Hospital of Zhengzhou University. In this study, none of the participants presented with hypertension, cardiopathy, diabetes, or renal dysfunction. All experiments using human samples were performed in accordance with the Declaration of Helsinki and the approval of the Institutional Review Board of Zhengzhou University.

### Animals and surgery

Male Wistar rats (200–250 g) were obtained from the Animal Center of Zhengzhou University and were kept in standard plastic cages. During one week of acclimatization, rats were randomly distributed in pairs per cage. They were maintained on *ad libitum* food and water with 12/12 h light/dark cycle. All animal experiments were performed in accordance with the National Institutes of Health Guide for the Care and Use of Laboratory Animals and approved by the Animal Ethics Committee of the Medical School of Zhengzhou University.

After one week of acclimatization, rats were randomly divided into four groups, (1) control rats with the sham operation and vehicle injection (Con), (2) 2VO surgery and vehicle injection (CCH), (3) 2VO surgery with *anta-miR-9-5p* injection (CCH+Anta-miR-9-5p), and (4) anta-miR-9-5p injection alone (Anta-miR-9-5p). For the 2VO surgery, rats were anesthetized with chloral hydrate (300 mg/kg, intraperitoneal [i.p.]), and bilateral common carotid arteries were gently separated from the carotid sheath and vagal nerves. Each artery was ligated with a 6-0 silk suture. Sham-operated controls received the same operation without ligation. After the procedure, rats were placed on a heating pad until recovery from anesthesia to maintain the body temperature at 37.5 ± 0.5 °C [23]. The antagomir of miR-9-5p (200 nM in aCSF) was administered through stereotaxic brain injection to the DG area (AP -2.0 mm, ML 1.5 mm, DV 2.0 mm) once at3 monthsafter the surgery. Sham-operated and controlled 2VO animals received the same volume of scrambled control or vehicle. The mortality was 1/17 (5.9%) in the Con group, 3/19 (15.8 %) in the CCH group, 2/15 (13.3 %) in the CCH+Anta-miR-9 group, and 1/14 (7.1 %) in the Anta-miR-9 group. Mortality rates did not differ significantly among the four groups.

### RNA isolation and miRNA detection

Total RNA was extracted from the cells and hippocampi using the Trizol Reagent (Invitrogen) according to the manufacturer's instructions. Real-time PCR reactions were performed using an ABI 7500 real-time PCR system (Applied Biosystems, CA, USA). Reverse transcription of the extracted miRNA was performed with miRNA-specific primers using the miRcute miRNA first-strand cDNA synthesis kit, and real-time PCR of miRNAs was performed using the miRcute miRNA qPCR detection kit according to the manufacturer's protocol (TIANGEN BIOTECH, China). U6 was used as endogenous controls and non-neoplastic brain tissues were used for calibration. The primers for detection of human (miRQ0000441-1-1) and rat (miRQ0000142-1-1) miR-9-5pwere provided by RiboBio Co., Ltd. (Guangzhou, China) and the primer for U6 are followed: sense: 5’-CTCGCTTCGGCAGCACA; anti-sense: 5’-AACGCTTCACGAATTTGCGT).

### Morris water maze

The Morris water maze (MWM) task was carried out at 3 months post-surgery [39, 40]. The maze consisted of a circular water tank (120 cm in diameter and 60 cm in depth) that was filled to a depth of 32 cm with opaque water and maintained at 22–26 °C. A hidden circular platform (10 cm in diameter) was submerged approximately 1.5 cm below the surface of the water and was kept at the same location in the southwest quadrant throughout the training period. All rats (including the sham-operated group) were subjected to daily MWM tests after completing a visual acuity test, as described previously [41]. Spatial training of the hidden platform in the water maze was performed for 6 consecutive days. Each rat received two trials per day with an intertrial interval of 1 min. The starting position (east, west, south, or north) for each trial was pseudo-randomly chosen and counterbalanced across all experimental groups. The swimming paths of the rats were monitored by a video camera linked to a computer. For each training trial, the latency to escape onto the hidden platform was recorded. The rats were given a maximum of 60 s to find the hidden platform. If the rat failed to find the platform within 60 s, the training was terminated and a maximum score of 60 s was assigned. Each rat was placed on the platform for 30 s before being removed from the water maze. For the probe trial on the 9th day of training [42], rats were subjected to a single 60-s swim without a hidden platform during the whole task, and the following durations were recorded: (1) time spent in the target quadrant where the platform had been placed during training, (2) the crossing times to the platform region, and (3) the initial time to cross the platform region. Swimming speed was also analyzed to evaluate motor ability [43].

### Step-down inhibitory avoidance task

Animals were subjected to training and test sessions in a step-down inhibitory avoidance task with an interval of 24 h. This task involves learning not to stepdown from a platform in order to avoid a mild foot shock. The inhibitory avoidance apparatus was an acrylic box (30 cm × 30 cm × 30 cm) with a floor consisting of parallel stainless steel bars (5 mm diameter) spaced 1 cm apart. A platform (5-cm wide × 5-cm high) was placed at one corner on the floor. Rats were placed on the platform and the time latency to step-down on the grid with all four paws was measured with an automatic device. During the training sessions, immediately after stepping down on the grid, the animals received an electrical foot shock (0.4 mA, 1.0 s scrambled). During the test sessions, no foot shock was delivered and step-down latency (with a ceiling of 180 s) was used as a measure of memory retention, as described in previous reports. The test sessions were performed 24 h after training to evaluate long-term memory [44, 45].

### Long-term potentiation recording

The long-term potentiation (LTP) recording and assay methods were performed as previously described [46]. Briefly, animals were anesthetized with chloral hydrate (300 mg/kg, i.p.) and placed on a stereotaxic instrument. The stimulating electrode was placed in the perforant path (anterior-posterior [AP] -7.0mm, mediolateral [ML] 4.5mm, dorsoventral [DV] 3.5 mm) and the recording electrode in the dentate gyrus region (AP -2.0 mm, ML 1.5 mm, DV 2.0 mm) of the hippocampus. The initial baseline responses were obtained by delivering a single pulse of stimulation once every 10 s. For each recording experiment, a stable baseline for at least 30 min was required before the application of conditioning stimuli. LTP was elicited using high-frequency stimulation (HFS) consisting of four trains of 50 pulses delivered at 200 Hz with a 2 s intertrain interval. The slope of the excitatory postsynaptic potential (EPSP) was recorded for 60 min and calculations were done by a computerized program (RM6240BD; Chengdu, China).

### Golgi staining

Golgi staining was performed as previously described [47]. Briefly, tissue slices (~4-mm thick) were placed in a solution containing 5% chloral hydrate, 5% potassium dichromate, 10% formalin in ddH_2_O for 3 days and then subjected to 1% silver nitrate under continuous vacuum for 4 days. Subsequently, brain tissues were cut into 40-μm thick sections with a vibratome and analyzed using a light microscope (Olympus BX60, Tokyo, Japan) with a 100X objective lens. The number of dendritic spines and the percent of mushroom-like spines on hippocampal DG pyramidal neurons were analyzed blindly (i.e. without knowledge of the treatment groups). For each experimental group, a minimum of 40 cells per animal (n = 4) were analyzedbyImage-Pro Plus 6.0 software. The criteria for the spine analysis were defined according to a previous study [48].

### ELISA

Twenty-four hours after the behavioral testing, rats were sacrificed under deep anesthesia. The hippocampi were subsequently dissected on ice and stored at -80 °C for further biochemical assays. The hippocampal samples were prepared as previously described [49]. For the analysis of the cholinergic function, the activity of choline acetyltransferase (ChAT) and acetylcholinesterase (AChE) was measured spectrophotometrically using commercial assay kits according to the manufacturer's instructions (Cat. No. A079-1, A024, Nanjing Jiancheng Biotech. Inc., Nanjing, China). The level of ACh in the hippocampalsupernatant was detected with an ELISA kit according to the manufacturer's instructions (Cat. No. A105-1, Nanjing Jiancheng Biotech. Inc., Nanjing, China). To measure oxidative stress, the activities of superoxide dismutase (SOD; Cat. No. A001-3), glutathione peroxidase (GSH-Px; Cat. No. A005), reactive oxygen species (T-ROS; Cat. No. E004), and the levels of malonic dialdehyde (MDA; Cat. No. A003-1) were examined using commercial kits (Nanjing Jiancheng Biotech., Inc., Nanjing, China).

### Nissl staining

The rats were anesthetized with an i.p. overdose of chloral hydrate and then perfused transcardially with 0.9% Sodium Chloride at 4 °C followed by 4% paraformaldehyde in 0.1 M phosphate-buffer (PB, pH 7.4). The whole brains were removed and postfixed in 4% paraformaldehyde at 4 °C for another 24 h. After dehydration in 30% and 40% sucrose until sunk, the brains were rapidly frozen in isopentane and 25-μm thick coronal sections were cut on a cryostat (CM 1950, Leica, Heidelberger, Germany). All the sections were used for Nissl staining, which was performed with 0.1% cresyl violet (Sigma, St Louis, MO, USA) to evaluate neuronal damage in the hippocampus. For cell counting, the NIH Image J software was used as previously reported [50].

### Statistical analysis

All data were expressed as the mean ± standard deviation (SD). Data were analyzed using two-way analysis of variance (ANOVA) followed by Duncan's multiple range test when appropriate. *P* values less than 0.05 (*P* < 0.05) were considered statistically significant.
